# Role of phosphodiesterase 4 expression in the Epac1 signaling‐dependent skeletal muscle hypertrophic action of clenbuterol

**DOI:** 10.14814/phy2.12791

**Published:** 2016-05-20

**Authors:** Yoshiki Ohnuki, Daisuke Umeki, Yasumasa Mototani, Kouichi Shiozawa, Megumi Nariyama, Aiko Ito, Naoya Kawamura, Yuka Yagisawa, Huiling Jin, Wenqian Cai, Kenji Suita, Yasutake Saeki, Takayuki Fujita, Yoshihiro Ishikawa, Satoshi Okumura

**Affiliations:** ^1^ Department of PhysiologyTsurumi University School of Dental MedicineYokohamaJapan; ^2^ Department of OrthodonticsTsurumi University School of Dental MedicineYokohamaJapan; ^3^ Department of Pediatric DentistryTsurumi University School of Dental MedicineYokohamaJapan; ^4^ Department of PeriodontologyTsurumi University School of Dental MedicineYokohamaJapan; ^5^ Cardiovascular Research InstituteYokohama City University Graduate School of MedicineYokohamaJapan

**Keywords:** Beta‐adrenoceptor, hypertrophy, signal transduction

## Abstract

Clenbuterol (CB), a selective *β*_2_‐adrenergic receptor (AR) agonist, induces muscle hypertrophy and counteracts muscle atrophy. However, it is paradoxically less effective in slow‐twitch muscle than in fast‐twitch muscle, though slow‐twitch muscle has a greater density of *β*‐AR. We recently demonstrated that Epac1 (exchange protein activated by cyclic AMP [cAMP]1) plays a pivotal role in *β*_2_‐AR‐mediated masseter muscle hypertrophy through activation of the Akt and calmodulin kinase II (CaMKII)/histone deacetylase 4 (HDAC4) signaling pathways. Here, we investigated the role of Epac1 in the differential hypertrophic effect of CB using tibialis anterior muscle (TA; typical fast‐twitch muscle) and soleus muscle (SOL; typical slow‐twitch muscle) of wild‐type (WT) and Epac1‐null mice (Epac1KO). The TA mass to tibial length (TL) ratio was similar in WT and Epac1KO at baseline and was significantly increased after CB infusion in WT, but not in Epac1KO. The SOL mass to TL ratio was also similar in WT and Epac1KO at baseline, but CB‐induced hypertrophy was suppressed in both mice. In order to understand the mechanism involved, we measured the protein expression levels of *β*‐AR signaling‐related molecules, and found that phosphodiesterase 4 (PDE4) expression was 12‐fold greater in SOL than in TA. These results are consistent with the idea that increased PDE4‐mediated cAMP hydrolysis occurs in SOL compared to TA, resulting in a reduced cAMP concentration that is insufficient to activate Epac1 and its downstream Akt and CaMKII/HDAC4 hypertrophic signaling pathways in SOL of WT. This scenario can account for the differential effects of CB on fast‐ and slow‐twitch muscles.

## Introduction

Skeletal muscle contains *β*‐adrenergic receptors (*β*‐AR), consisting of about 90% *β*_2_‐subtype and approximately 10% *β*_1_‐subtype, together with a smaller population of *α*‐AR, which is usually found in higher proportions in slow‐twitch muscles (Williams et al. 1984; Rattigan et al. 1986). Clenbuterol (CB), a selective *β*_2_‐AR agonist, induces muscle hypertrophy and counteracts unloading‐induced (Ricart‐Firinga et al. 2000) or dexamethasone‐induced muscle atrophy (Jiang et al. 1996) by increasing muscle protein synthesis or decreasing protein degradation, or both (Lynch and Ryall 2008). However, the molecular mechanisms underlying its anabolic effects on skeletal muscle are not fully understood.

Recently, we developed a mouse model, in which exchange protein directly activated by cyclic AMP 1 (Epac1), a major skeletal muscle isoform, was disrupted (Epac1KO) (Okumura et al. 2014). In this mouse model, CB‐induced hypertrophy of masseter muscle, which is composed of predominantly fast‐twitch fibers, was abolished, but myosin heavy chain (MHC) isoform transition toward faster isoforms was induced in the same manner as in wild‐type (WT) controls. We also demonstrated attenuation of the Epac1‐mediated activation of Akt and its downstream target mTOR (originally designed as “mammalian target of rapamycin,” but now officially called “mechanistic target of rapamycin”) (Laplante and Sabatini 2012), that is, the Akt/mTOR pathway, as well as the calmodulin kinase II (CaMKII)/histone deacetylase 4 (HDAC4) pathway (Ohnuki et al. 2014). Epac activation has been demonstrated to induce nuclear efflux of HDAC4 through CaMKII‐mediated phosphorylation on serine 246, with consequent activation of a prohypertrophic transcription factor, that is, myocyte enhancer factor 2 (MEF2), in skeletal muscle (Liu and Schneider 2013; Ohnuki et al. 2014), as demonstrated previously in cardiac myocytes (Metrich et al. 2010). In addition to serving as a repressor of MEF2 transcriptional activity, HDAC4 induces transcription of ubiquitin E3 ligases atrogin‐1 and MuRF1, which promote muscle atrophy by increasing myogenin expression (Moresi et al. 2010) or by activating mitogen‐activated protein kinase/activator protein‐1 signaling (Choi et al. 2012) in skeletal muscle, implying that CaMKII‐dependent nuclear efflux of HDAC4 could inhibit protein degradation by suppressing transcription of atrogin‐1 and MuRF1. We thus proposed that loss of Epac1‐mediated activation of these downstream signaling pathways might be the key event in the blockade of CB‐induced masseter muscle hypertrophy in Epac1KO (Ohnuki et al. 2014).

Slow‐twitch muscles have a greater density of *β*‐AR than fast‐twitch muscles (Chen and Alway 2001; Ryall et al. 2002, 2006). Although the physiological relevance of this difference is unclear, CB is paradoxically less effective in inducing hypertrophy of slow‐twitch muscle, such as soleus muscle (SOL) (Ryall et al. 2002), compared to fast‐twitch muscles, such as tibialis anterior muscle (TA) (Shi et al. 2007), extensor digitorum longus muscle (Ryall et al. 2002; Shi et al. 2007), and masseter muscle (Ohnuki et al. 2014). A recent study also supported the notion of the paradoxical less hypertrophic effect of CB on SOL, compared to the extensor digitorum longus muscle (Py et al. 2015). Since *β*‐AR signaling represents a therapeutic target for the management of skeletal muscle wasting and weakness, it is important to understand the mechanisms underlying the differential hypertrophic effect of CB on fast‐ and slow‐twitch muscles (Kissel et al. 1998; Lynch and Ryall 2008). We hypothesized that the difference of the hypertrophic effects of CB on the two types of muscle might be due to a difference in *β*_2_‐AR downstream signaling. In the present work, we examined this hypothesis using our Epac1KO mouse model and WT controls.

## Methods

### Mice and experimental protocol

We have previously reported the generation of Epac1KO (ACC. No. CDB0542K: http://www.cdb.riken.jp/arg/mutant%20mice%20list.htm) (Suzuki et al. 2010; Ohnuki et al. 2014; Okumura et al. 2014). All experiments were performed on C57BL/6 and CBA mixed‐background homozygous Epac1KO (6.8 ± 0.3‐month‐old, *n *=* *12) and their WT littermates (6.4 ± 0.4‐month‐old, *n *=* *12). This study was approved by the Animal Care and Use Committees of Tsurumi University and Yokohama City University School of Medicine.

Clenbuterol (Sigma, St. Louis, MO) was dissolved in saline to prepare a 0.6 mg/mL stock solution and the appropriate volume of this solution to provide the desired dose (2 mg/kg) was added to 0.2 mL of saline to prepare the solution for intraperitoneal (i.p.) injection (Pearen et al. 2009; Goodman et al. 2011; Ohnuki et al. 2014). CB was administered i.p. once daily for 3 weeks, and control mice received an identical volume of saline only in both WT and Epac1KO.

**Figure 1:**
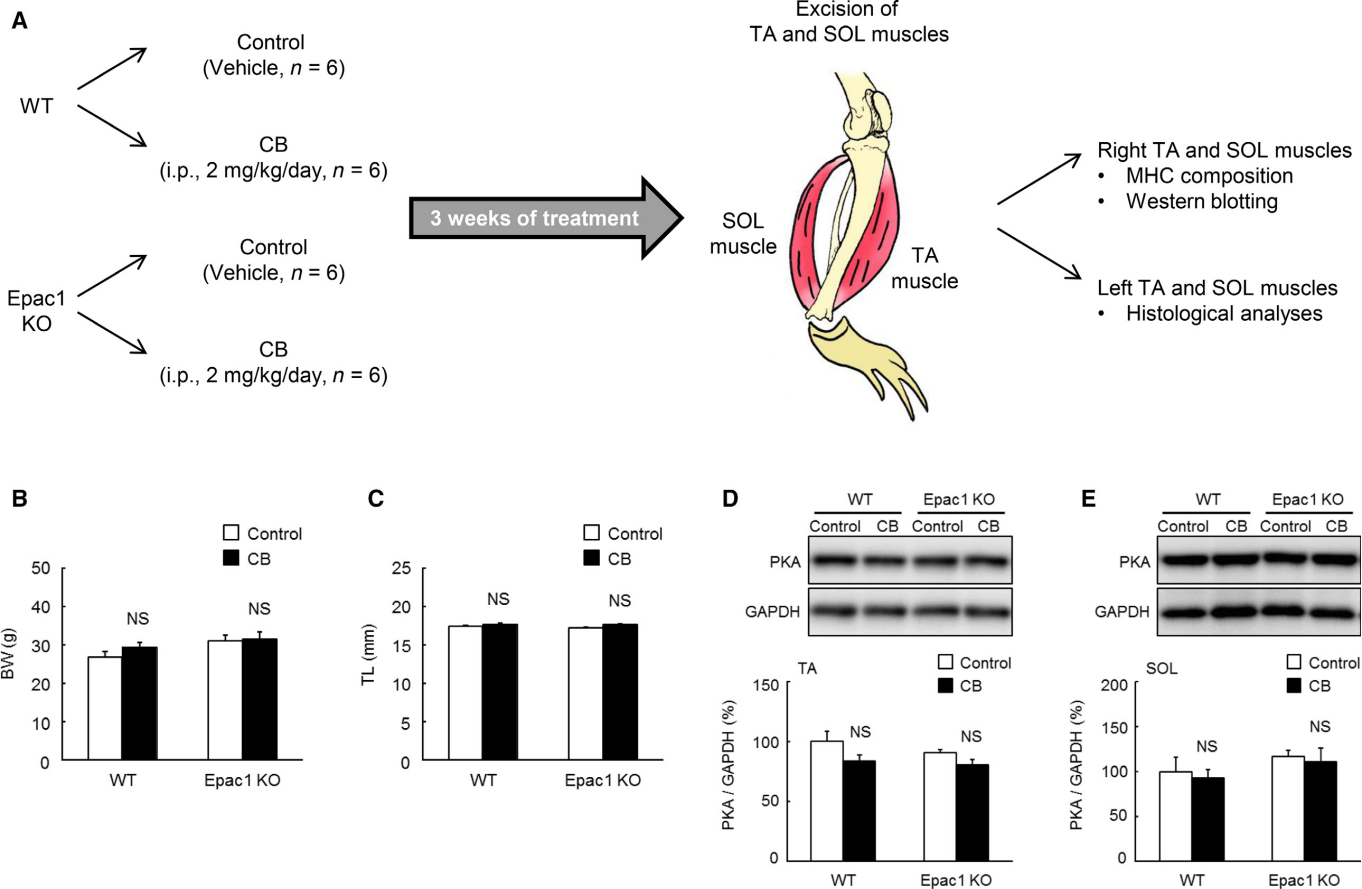
Experimental procedures and effects of CB on body weight, tibial length, and PKA expression in Epac1KO. (A) Clenbuterol (CB) was administered once daily for 3 weeks via intraperitoneal injection (i.p.) at a dose of 2 mg/kg, dissolved in saline. Age‐matched control mice (Control) received an identical volume of saline only. (B and C), Body weight (BW; g) and tibial length (TL; mm) of Control and CB‐treated WT and Epac1KO. No significant difference in BW (B) or TL (C) was observed between Control and CB‐treated WT or Epac1KO (*P *= NS vs. Control by Tukey's test, *n *=* *6 each). (D and E), PKA expression levels (total PKA‐catalytic units) in TA and SOL. No significant difference of PKA expression in either TA (D) or SOL (E) was observed between Control and CB‐treated WT or Epac1KO (*P *= NS vs. Control by Tukey's test, *n *=* *6 each). The amount of expression in WT treated with saline (Control) was taken as 100% in each determination and representative immnunoblotting results are shown for total PKA catalytic units and GAPDH.

The dose of CB used in this study has been reported to increase skeletal muscle mass without affecting body weight (Ryall et al. 2002). After completion of each treatment, mice were anesthetized with isoflurane and TA and SOL muscles were excised from the right and left legs. The specimens were weighed, frozen in liquid nitrogen, and stored at −80°C for later analysis (Fig. 1A). The muscle mass to tibial length (TL; mm) ratio was used as indexes of muscle growth. After tissue extraction, the mice were killed by cervical dislocation (Goodman et al. 2011).

### Histological analysis

Cross sections (10 *μ*m thick) were cut from the middle portion of the left TA and SOL muscles with a cryostat (CM1900, Leica Microsystems, Nussloch, Germany) at −20°C. The sections were stained with hematoxylin and eosin (HE) and observed under a light microscope (BX61, Olympus Co., Tokyo, Japan) (Okumura et al. 2014). Micrographs were taken with a digital camera (DP‐72, Olympus Co.) connected to a personal computer. The cross‐sectional size of muscle fibers was evaluated by measuring the cross‐sectional area (CSA) of 100 muscle fibers with image analysis software (Image J 1.45) and averaged to obtain the mean value in each mouse (Umeki et al. 2015).

### MHC composition

Myosin heavy chain isoform composition in TA and SOL muscles excised from the right legs (Fig. 1A) was analyzed by means of SDS‐PAGE, followed by silver staining of the MHC isoform bands (Silver Staining Kit, GE Healthcare, Buckinghamshire, UK). The stained bands were scanned with a densitometer (LAS‐1000, Fuji Photo Film, Tokyo, Japan). To determine MHC composition, the relative proportion of each MHC isoform was calculated as a percentage of total MHC content using the integrated dye density of the bands (Ohnuki et al. 2013, 2014; Umeki et al. 2015).

### Western blotting

Tibialis anterior or soleus muscle excised from the right leg (Fig. 1A) was homogenized in a Polytron (Kinematica AG, Lucerne, Switzerland) in ice‐cold RIPA buffer (Thermo Fisher Scientific, Waltham, MA: 25 mmol/L Tris‐HCl (pH 7.6), 150 mmol/L NaCl, 1% NP‐40, 1% sodium deoxycholate, 0.1% SDS) without addition of inhibitors (Yu et al. 2011), and the homogenate was centrifuged at 13,000 × *g* for 10 min at 4°C. The supernatant was collected and the protein concentration was measured using a DC protein assay kit (Bio‐Rad, Hercules, CA). Equal amounts of protein (5 *μ*g) were subjected to 12.5% SDS‐polyacrylamide gel electrophoresis and blotted onto 0.2 *μ*m PVDF membrane (Bio‐Rad).

Western blotting was conducted with commercially available antibodies (Okumura et al. 2003a,b, 2008, 2009; Bai et al. 2012). The primary antibodies against CREB (#9197), phospho‐CREB (Ser‐133, #9198), Akt (#9272), phospho‐Akt (Ser‐473, #9271), 70‐kDa ribosomal S6 kinase 1 (S6K1) (#9202), phospho‐S6K1 (Thr‐389, #9205), CaMKII (#3362), phosphor‐CaMKII (Thr‐286, #3361), HDAC4 (#7628), phosphor‐HDAC4 (Ser‐246, #3443), Epac1 (#4155) and Epac2 (#4156) were purchased from Cell Signaling Technology (Boston, MA) and the primary antibodies against *β*_2_‐AR (sc‐569), protein kinase A (PKA)‐catalytic subunit (sc‐903), phosphor‐PKA‐catalytic subunit (Thr‐198, sc‐32968), PDE4D (sc‐25814), and glyceraldehyde 3‐phosphate dehydrogenase (GAPDH) (sc‐25778) were purchased from Santa Cruz Biotechnology (Santa Cruz, CA). Horseradish peroxidase‐conjugated anti‐rabbit IgG (NA934; GE Healthcare) or anti‐mouse IgG (NA931; GE Healthcare) was used as a secondary antibody. The primary and secondary antibodies were diluted in Tris‐buffered saline (pH 7.6) containing 0.1% Tween 20 and 5% bovine serum albumin. The blots were visualized with enhanced chemiluminescence solution (ECL Prime Western Blotting Detection Reagent, GE Healthware) and scanned with a densitometer (LAS‐1000, Fuji Photo Film).

### Immunohistochemical staining

The specimens were embedded in Tissue‐Tek OCT compound (Miles Laboratories, Elkhart, IN) and frozen in liquid nitrogen. Cross sections (10 *μ*m thick) were cut from the middle portion of the specimens with a cryostat at −20°C and immunohistochemical staining were performed with monoclonal antibodies against skeletal type II (fast‐type) (MY‐32; Sigma) and type I (slow‐type) (NOQ7.5.4D; Sigma) myosin. The immunoreaction was visualized with the Vectastain Universal Elite ABC kit (PK‐6200; Vector Laboratories, Burlingame, CA) and AEC Substrate Kit (SK‐4200, Vector Laboratories), and observed under a light microscope (Nikon, Tokyo, Japan).

### Measurement of cAMP levels

cAMP levels in TA or SOL of WT and Epac1KO were measured after treatment with CB (2 mg/kg dissolved in 0.2 mL saline) or saline alone as a control. After 1 hour, mice were killed by cervical dislocation, and the TA and SOL muscles were removed immediately. Connective tissue was trimmed away; then, the samples were placed in liquid nitrogen and stored at −80°C. cAMP levels in TA and SOL muscles were measured within 24 h using cAMP EIA System (RPN2251, GE Healthcare) according to the manufacturer's protocol.

### Statistical analysis

**Figure 2:**
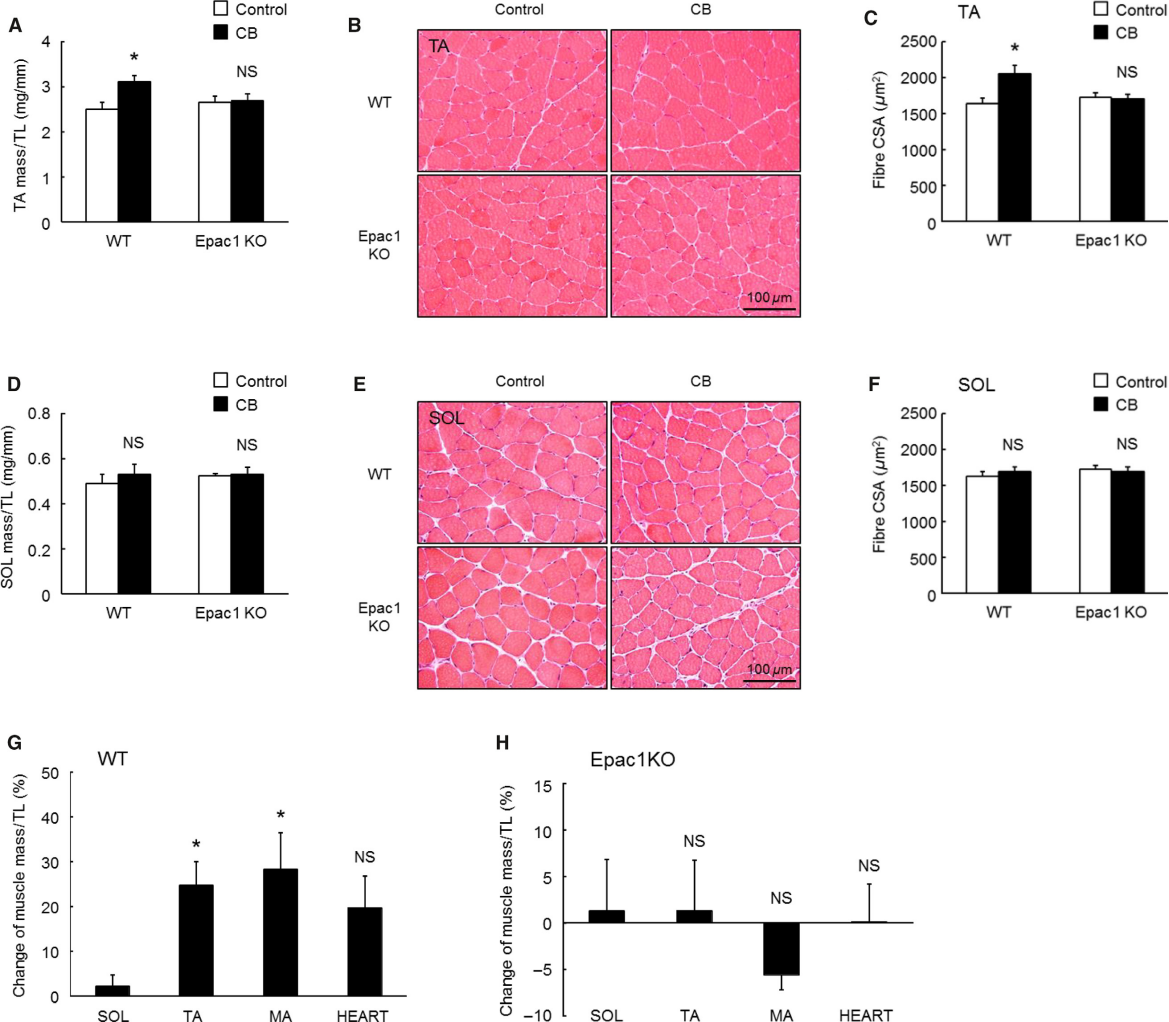
Effect of CB on muscle mass/TL ratio, histological analysis, and change from baseline of mass/TL ratio of TA, SOL, masseter muscle, and cardiac muscle in response to chronic CB infusion. Muscle mass (mg)/tibial length (TL; mm) ratio (A and D), cross‐sections (B and E), fiber cross‐sectional area (CSA;* μ*m^2^) (C and F) of TA (A–C) or SOL (D–F) prepared from Control and CB‐treated WT and Epac1KO. Changes from baseline of muscle mass (TA, SOL, masseter muscle (MA) and cardiac muscle (HEART)) (mg)/TL (mm) ratio (%) in response to chronic CB infusion in WT (G) and Epac1KO (H). (A–C), TA mass/TL ratio (A) were significantly increased by CB treatment in WT (*P <* *0.05 by Tukey's test, *n *=* *6), but not in Epac1KO (*P* = NS by Tukey's test, *n *=* *6). No abnormality of TA muscle organization was observed in Control or CB‐treated WT or Epac1KO (B). Fiber CSA was significantly increased by CB treatment in WT (*P <* *0.05 by Tukey's test, *n *=* *6), but not in Epac1KO (*P* = NS by Tukey's test, *n *=* *6) (C). (D–F), SOL mass/TL ratio (D) were not significantly different between Control and CB‐treated WT or Epac1KO (*P* = NS vs. Control by Tukey's test, *n *=* *6). No abnormality of SOL muscle organization was observed in Control or CB‐treated WT or Epac1KO (E). Fiber CSA was not significantly different between Control and CB‐treated WT or Epac1KO (*P* = NS vs. Control by Tukey's test, *n *=* *6 each) (F). (G) The change in mass/TL ratio (%) of SOL was significantly smaller than that of TA, MA or HEART mass (*P <* *0.05 vs. SOL muscle by Tukey's test, *n *=* *6 each). (H) Change of mass/TL ratio (%) of SOL was suppressed in Epac1KO (*P *= NS vs. SOL muscle by Tukey's test, *n *=* *5–6).

**Figure 3:**
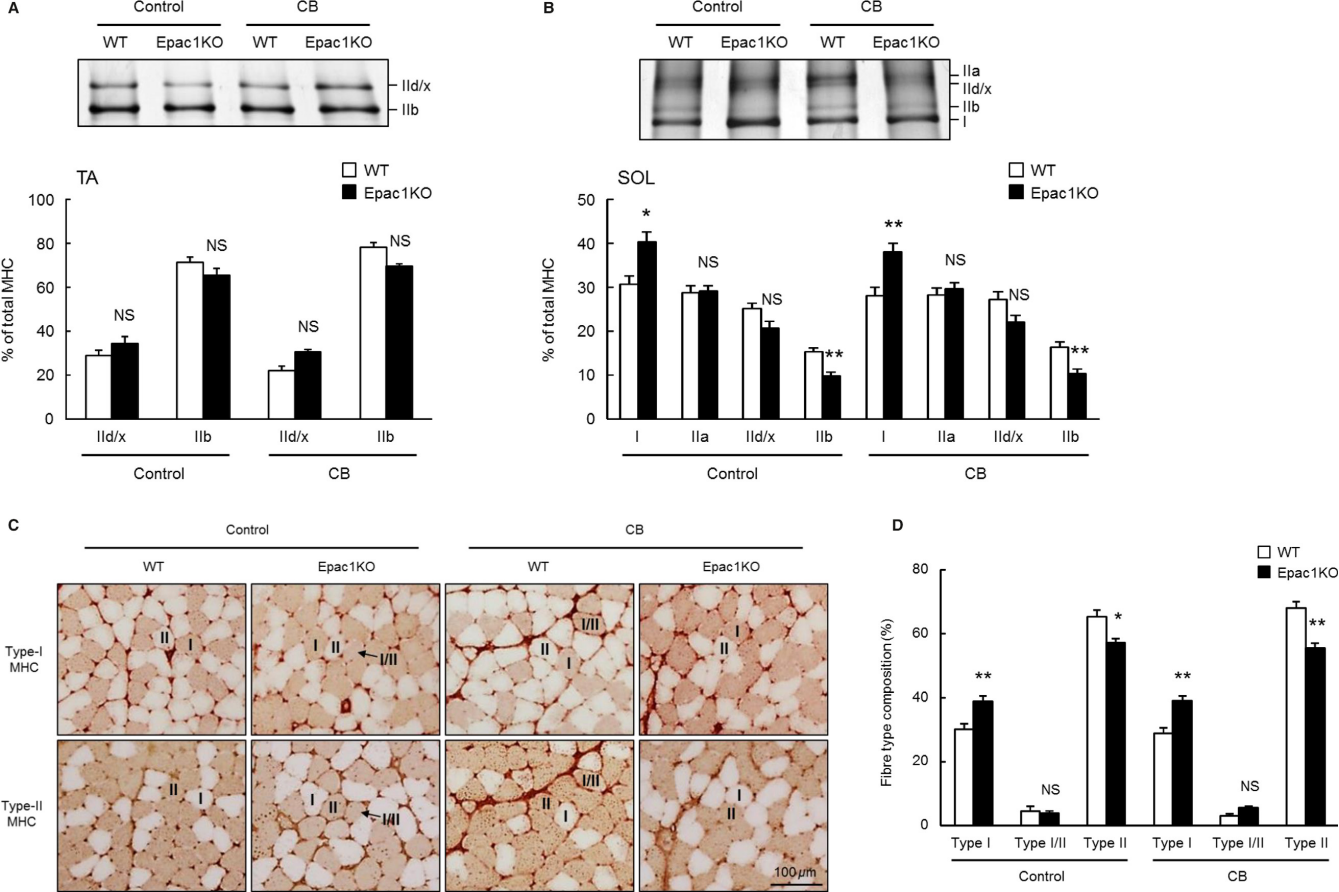
Effects of CB on MHC and fiber‐type composition in TA and SOL muscles. Typical SDS‐PAGE profiles of MHC isoforms (*upper*) and the average MHC composition (*lower*) of TA (A) and SOL (B) prepared from Control and CB‐treated (CB) WT and Epac1KO. The average proportion of each MHC isoform was expressed as a percentage of total MHC content (% of total MHC). (A) The proportions of MHC‐IId/x and MHC‐IIb in TA muscle were not significantly different between WT and Epac1KO in either the Control or CB‐treated group (*P* = NS vs. WT by Tukey's test, *n *=* *6). (B) The proportion of MHC‐I in SOL prepared from Epac1KO was significantly greater than that of WT in both the Control and CB‐treated groups (*P <* *0.05, **P <* *0.01 vs. WT by Tukey's test, *n *=* *6), while that of MHC‐IIb in SOL prepared from Epac1KO was significantly smaller than that of WT in both the Control and CB‐treated groups (**P <* *0.01 vs. WT by Tukey's test, *n *=* *6). The proportions of MHC‐IIa and MHC‐IId/x were not significantly different between WT and Epac1KO in either the Control or CB‐treated group (*P* = NS vs. WT by Tukey's test, *n *=* *6). (C) Representative cross sections of immunohistochemical staining for type I (*upper*) and type II (*lower*) fibres in SOL prepared from Control (*left*) and CB‐treated (CB) (*right*) WT and Epac1KO. (D) Quantitative comparison of fiber type composition in SOL between WT and Epac1KO in the Control and CB‐treated groups. The proportion of type I fiber in Epac1KO was significantly greater than that in WT (**P <* *0.01 vs. WT by Tukey's test, *n *=* *6), while that of type II fiber in Epac1KO was significantly smaller than that in WT in both the Control and CB‐treated groups (*P <* *0.05, **P <* *0.01 vs. WT by Tukey's test, *n *=* *6). The proportion of intermediate type (Type I/II) was not significantly different between WT and Epac1KO in either the Control or CB‐treated groups (*P* = NS vs. Control by Tukey's test, *n *=* *6). The average proportion of fiber type was expressed as a percentage of total fiber number (% of total fiber).

**Figure 4:**
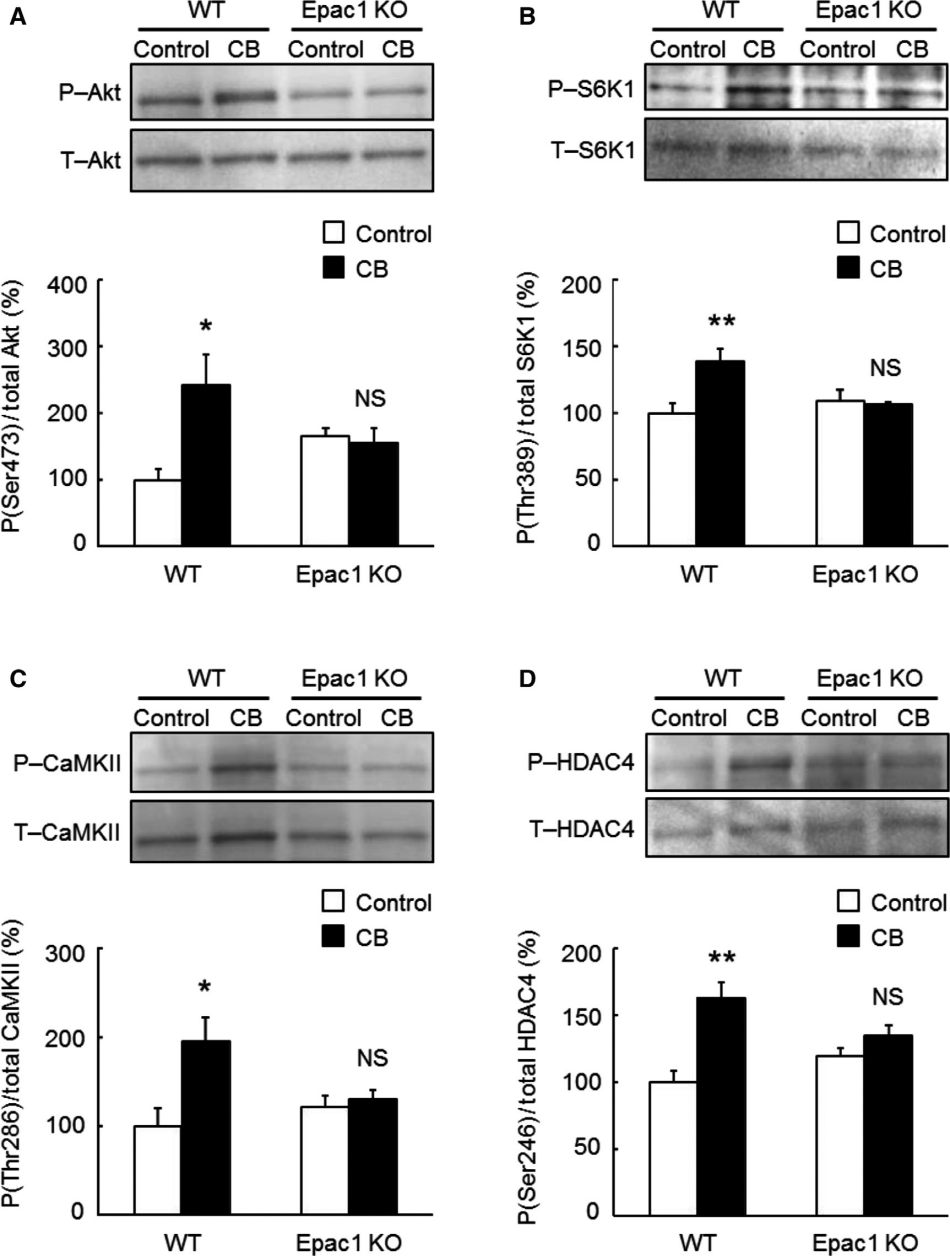
Activation of Akt/mTOR signaling or CaMKII/HDAC4 signaling in TA muscle of WT and Epac1KO in response to chronic CB treatment. (A–B) Activation of Akt/mTOR signaling was examined by measuring phosphorylated and total Akt (A) and S6K1 (B) in TA of WT and Epac1KO after chronic CB treatment for 3 weeks. Significant increases of phosphorylated Akt (Ser 476) and S6K1 (Thr 389) molecules were observed in WT (*P <* *0.05, **P <* *0.01 vs. Control by Tukey's test, *n *=* *4–6), but not in Epac1KO (*P* = NS vs. Control by Tukey's test, *n *=* *5–6). (C–D) Phosphorylated and total CaMKII (C) and HDAC4 (D) in WT and Epac1KO after chronic CB infusion. Phosphorylation of both CaMKII (Thr 286) and HDAC4 (Ser 246) was significantly increased in WT (*P <* *0.05, **P <* *0.01 vs. Control by Tukey's test, *n *=* *5–6), but not in Epac1KO (*P* = NS vs. Control by Tukey's test, *n *=* *4–6). The amount of expression in WT treated with saline was taken as 100% in each determination and representative immnunoblotting results are shown for phosphorylated and total Akt, S6K1, CaMKII, and HDAC4.

**Figure 5:**
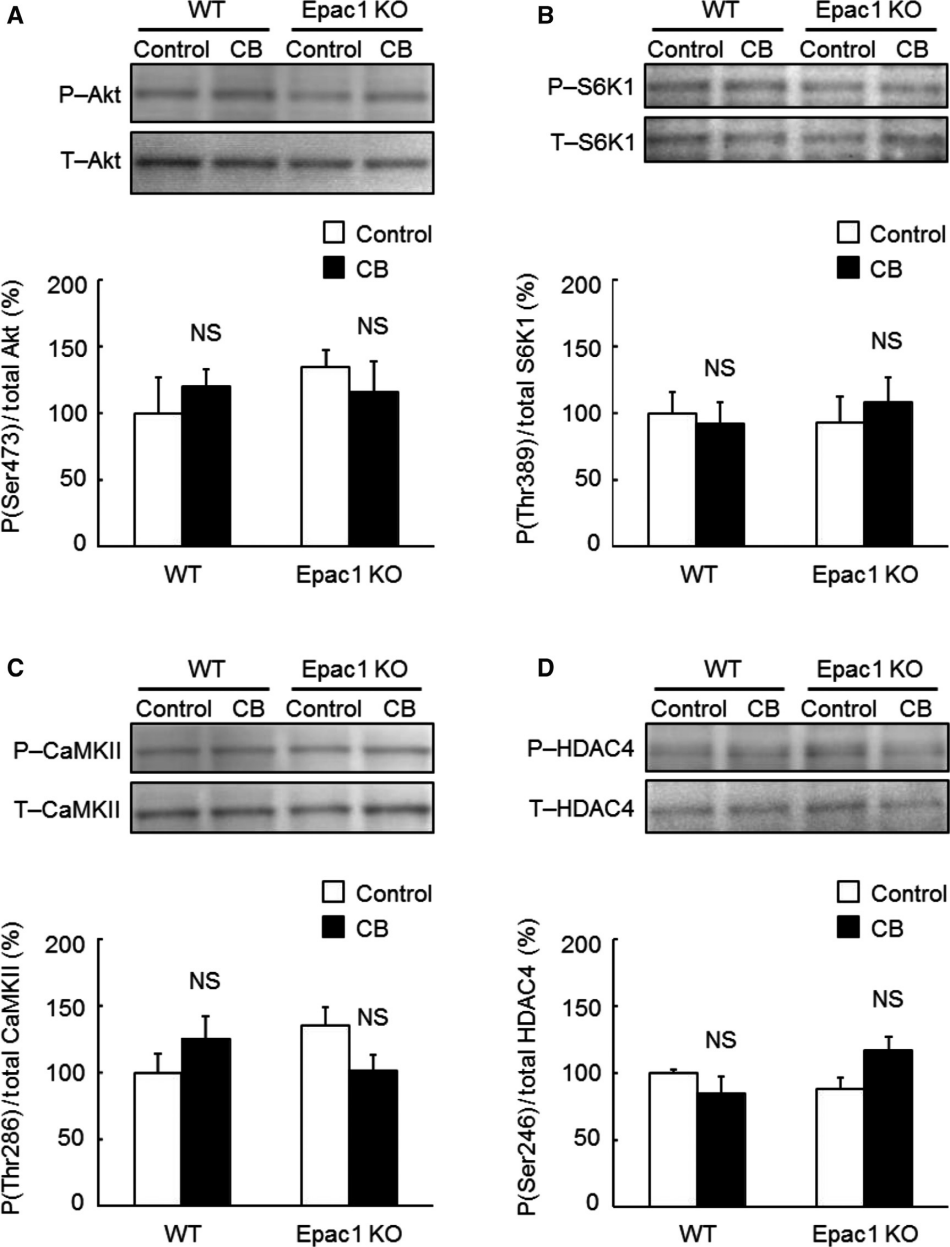
Activation of Akt/mTOR signaling or CaMKII/HDAC4 signaling in SOL muscle of WT and Epac1KO in response to chronic CB infusion. (A–B) Activation of Akt/mTOR signaling was examined by measuring phosphorylated and total Akt (A) and S6K1 (B) in SOL prepared from WT and Epac1KO after chronic CB treatment for 3 weeks. Significant increases of phosphorylated Akt (Ser 476) and S6K1 (Thr 389) molecules were not observed in either WT or Epac1KO (*P* = NS vs. Control by Tukey's test, *n *=* *6 each). (C–D) Phosphorylated and total CaMKII (C) and HDAC4 (D) in SOL of WT and Epac1KO after chronic CB infusion for 3 weeks. Significant increases of phosphorylated CaMKII (Thr 286) and HDAC4 (Ser 246) were not observed in either WT or Epac1KO (*P* = NS vs. Control by Tukey's test, *n *=* *6). The amount of expression in WT treated with saline was taken as 100% in each determination and representative immnunoblotting results are shown for phosphorylated and total Akt, S6K1, CaMKII, and HDAC4

**Figure 6:**
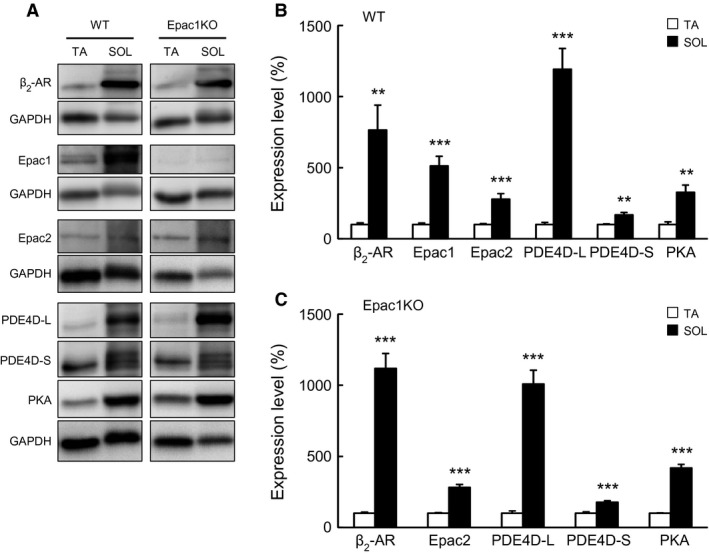
Expression of *β*_2_‐AR, Epac1/2, PDE4D and PKA in TA and SOL muscles prepared from WT and Epac1KO at baseline. (A) Representative immunoblotting results of total *β*_2_‐AR, Epac1/2, PDE4D‐L (long isoforms), PDE4D‐S (short isoforms), and PKA in TA and SOL prepared from WT or Epac1KO at baseline. (B) Expression of *β*_2_‐AR, Epac1/2, PDE4D‐L, PDE4D‐S, and PKA in TA muscle and SOL muscle prepared from WT at baseline. The expression of each molecule in SOL was significantly greater than that in TA (**P <* *0.01, ***P <* *0.001 vs. TA by unpaired *t*‐test, *n *=* *4–5), but the magnitude of the increase of PDE4D‐L was much greater (12‐fold), compared to *β*_2_‐AR (7.6‐fold), Epac1 (5.1‐fold), Epac2 (2.8‐fold) and PDE4D‐S (1.7‐fold) and PKA (3.3‐fold). The amount of expression in TA muscle of WT treated with saline (Control in WT) was taken as 100% in each determination. (C) Expression of *β*_2_‐AR, Epac1/2, PDE4D‐L, PDE4D‐S, and PKA in TA and SOL prepared from Epac1KO at baseline. The expression of each molecule in SOL was similarly and significantly greater than that in TA (**P <* *0.01 vs. TA by unpaired *t*‐test, *n *=* *4–5), but the magnitude of the increase of PDE4D‐L (11‐fold) was much greater, compared to those of *β*_2_‐AR (2.8‐fold), Epac2 (10.1‐fold), PDE4D‐S (1.8‐fold), and PKA (4.2‐fold). The amount of expression in TA muscle of Epac1KO treated with saline (Control in Epac1KO) was taken as 100% in each determination.

**Figure 7:**
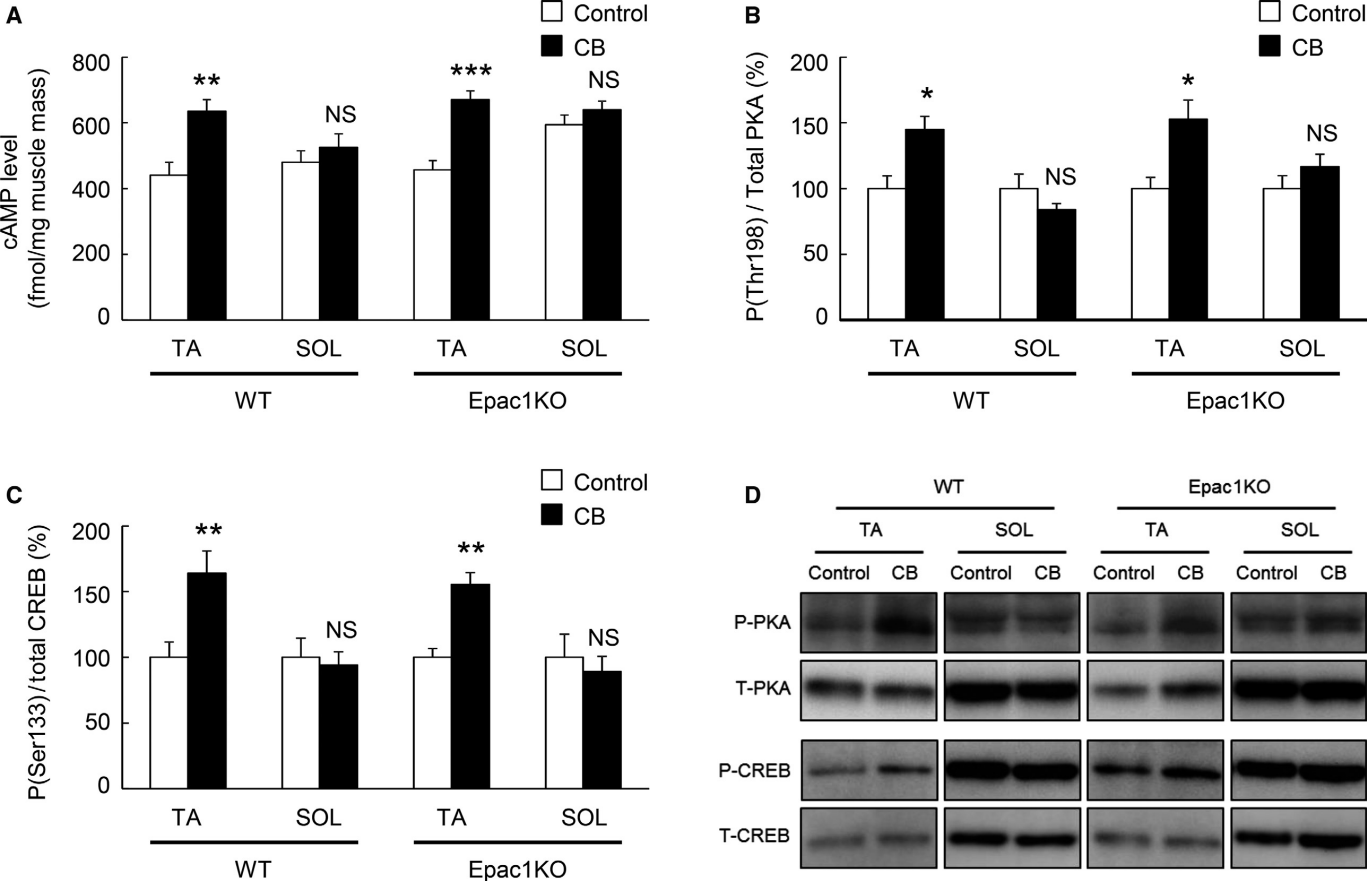
Activation of cAMP signaling was attenuated in SOL, compared to that in TA. (A) cAMP level was significantly increased in TA (**P <* *0.01, ***P <* *0.001 vs. Control by unpaired *t*‐test, *n *=* *6–7) but not in SOL muscle (*P* = NS vs. Control by unpaired *t*‐test, *n *=* *6–7) after CB treatment for 60 min in either WT (*left*) or Epac1KO (*right*). (B) Phosphorylation level of PKA‐catalytic unit (threonine 198) was significantly increased in TA prepared from WT (*left*) and Epac1KO (*right*) (*P <* *0.05 vs. Control by unpaired *t*‐test, *n *=* *5–6 each), but the increase was suppressed in SOL muscle (*P* = NS vs. Control by unpaired *t*‐test, *n *=* *5–6 each) after the CB treatment for 3 weeks. The amount of expression in WT treated with saline was taken as 100% in each determination. (C) Phosphorylation level of CREB (serine 133) was significantly increased in TA prepared from WT (*left*) and Epac1KO (*right*) (**P <* *0.01 vs. Control by unpaired *t*‐test, *n *=* *4–6 each), but the increase was suppressed in SOL muscle (*P* = NS vs. Control by unpaired *t*‐test, *n *=* *5–6 each) after CB treatment for 3 weeks. The amount of expression in WT treated with saline was taken as 100% in each determination. (D) Representative immunoblotting results of phosphorylated and total PKA and CREB in TA and SOL muscles prepared from WT (*left*) and Epac1KO (*right*) after chronic CB treatment for 3 weeks.

Data are expressed as means ± SEM. The statistical significance of differences was determined using Student's unpaired *t*‐test (Figs. 6B and C, 7A–C), one‐way ANOVA (Fig. 2G and H), or two‐way ANOVA (genotype and treatment main effects, and interaction effect) (Figs. 1B–E, 2A, C, D, F, 3A–B, D, 4, 5), as appropriate. Tukey's *post hoc* test was used to locate significant differences between the Control and CB‐treated groups in either WT or Epac1KO (Figs. 1B–E, 2A, C, D, F, 4, 5) or between WT and Epac1KO in either Control or CB‐treated group (Fig. 3A, B, D). The criterion of significance was taken as *P *<* *0.05.

## Results

### Effects of CB on body weight and tibial length

We first examined the effects of CB on BW (Fig. 1B, genotype and treatment main effect, and interaction effect, *P *= not significant (NS) by two‐way ANOVA). BW was not different between the Control and CB‐treated groups in either WT (27 ± 1.6 g (Control) vs. 29 ± 1.2 g (CB), *P *=* *NS by Tukey's test, *n *=* *6), and Epac1KO (31 ± 1.5 g (Control) vs. 32 ± 1.9 g (CB), *P* = NS by Tukey's test, *n *=* *6). We also examined the effects of CB on TL (Fig. 1C, genotype and treatment main effect, and interaction effect, *P *= NS by two‐way ANOVA) because Epac was recently reported to be involved in bone formation in vitro (Hutchings et al. 2009; Prideaux et al. 2015). However, TL was similar in the Control and CB‐treated groups (WT: 17 ± 0.1 mm (Control) vs. 18 ± 0.2 mm (CB), Epac1KO: 17 ± 0.1 mm (Control) vs. 18 ± 0.2 mm (CB), *P *= NS by Tukey's test, *n *=* *6).

### Effects of CB on PKA expression

We also examined the effect of CB on PKA expression in TA and SOL muscles by measuring the expression of total PKA‐catalytic units (Ohnuki et al. 2014; Okumura et al. 2014). In TA, there was no difference in expression level between the Control and CB‐treated groups in either WT (100 ± 8.6% (Control) vs. 84 ± 5.3% (CB), *P *=* *NS by Tukey's test, *n *=* *6) or Epac1KO (91 ± 2.4% (Control) vs. 80 ± 4.9% (CB), *P *=* *NS by Tukey's test, *n *=* *6) (Fig. 1D, significant treatment main effect, *P *<* *0.05 by two‐way ANOVA). In SOL, there was also no difference in expression level between the Control and CB‐treated groups in either WT (100 ± 15.6% (Control) vs. 93 ± 9.1% (CB), *P *=* *NS by Tukey's test, *n *=* *5–6) or Epac1KO (117 ± 6.9% (Control) vs. 111 ± 14.9% (CB), *P *=* *NS by Tukey's test, *n *=* *6) (Fig. 1E, genotype and treatment main effect, and interaction effect, *P *= NS by two‐way ANOVA). These data indicate that PKA expression in TA and SOL muscles was not altered in Epac1KO at baseline or after CB infusion, in accordance with findings in masseter muscle and cardiac muscle (Ohnuki et al. 2014; Okumura et al. 2014).

### CB‐induced TA muscle hypertrophy was suppressed in Epac1KO

The TA mass (mg) to TL (mm) ratio were significantly increased by CB treatment in WT (TA mass/TL: 2.5 ± 0.2 mg/mm (Control) vs. 3.1 ± 0.2 mg/mm (CB)*, P *<* *0.05 by Tukey's test, *n *=* *6). However, these increases were suppressed in Epac1KO (TA mass/TL: 2.7 ± 0.1 mg/g vs. 2.8 ± 0.6 mg/g (CB), (Control) *P *= NS by Tukey's test, *n *=* *6) (Fig. 2A, significant treatment main effect, *P *<* *0.05 by two‐way ANOVA).

Histological analysis showed no TA abnormality (such as fibrosis) in either the Control or CB‐treated WT or Epac1KO (Fig. 2B).

We also examined TA muscle hypertrophy in terms of CSA (Fig. 2C). CSA of the CB‐treated group was significantly greater than that of the Control in WT (CSA: 1639 ± 77 *μ*m^2^ (Control) vs. 2053 ± 119 *μ*m^2^ (CB), *P *<* *0.05 by Tukey's test, *n *=* *6), while CSA of the CB‐treated Epac1KO was similar to that of the Control (CSA: 1729 ± 61 *μ*m^2^ (Control) vs. 1706 ± 58 *μ*m^2^ (CB), *P *= NS by Tukey's test, *n* = 6) (Fig. 2C, significant treatment main effect, *P *<* *0.05 by two‐way ANOVA).

These data indicate that Epac1 plays an important role in the development of CB‐induced, *β*_2_‐AR‐mediated hypertrophy of TA.

### CB did not induce SOL muscle hypertrophy in either WT or Epac1KO

We next examined the effect of CB on SOL. The SOL mass (mg) to TL (mm) ratio was not significantly different between the Control and CB‐treated groups of WT (SOL mass/TL: 0.5 ± 0.04 mg/mm (Control) vs. 0.5 ± 0.04 mg/mm (CB), *P *= NS by Tukey's test, *n *=* *6) or Epac1KO (SOL mass/TL: 0.52 ± 0.01 mg/mm (Control) vs. 0.53 ± 0.03 mg/mm (CB), *P *= NS by Tukey's test, *n *=* *6) (Fig. 2D, genotype and treatment main effects, and interaction effect, *P *= NS by two‐way ANOVA). These results in WT are in marked contrast to those shown above for TA (Fig. 2A).

Histological analysis showed no abnormality of SOL muscle organization in either the Control or CB‐treated WT or Epac1KO (Fig. 2E).

Cross‐sectional area was not significantly different between WT and Epac1KO at baseline and they remained unchanged by CB treatment in both WT (CSA: 1632 ± 63 *μ*m^2^ (Control) vs. 1695 ± 60 (CB) *μ*m^2^, *P *= NS by Tukey's test, *n *=* *6) and Epac1KO (CSA: 1721 ± 60 *μ*m^2^ (Control) vs. 1693 ± 69 *μ*m^2^ (CB), *P *= NS by Tukey's test, *n *=* *6) (Fig. 2F, genotype and treatment main effects, and interaction effect, *P *= NS by two‐way ANOVA). Again, this is in marked contrast with the case of TA in WT.

We also compared the muscle mass to TL ratio (normalized as change from the Control value, %) of TA and SOL with that of masseter (MA) and cardiac muscles after CB infusion (Fig. 2G and H). The ratio for masseter muscle was significantly increased from baseline and the magnitude of the increase was not significantly different from that of TA (28 ± 8.3% (MA) vs. 25 ± 5.3% (TA), *P *= NS by Tukey's test, *n *=* *5–6). Cardiac muscle also showed a similar response (20 ± 7.1%, *P *= NS by Tukey's test, *n *=* *5) to TA. However, SOL showed a significantly decreased response to CB, compared to the other muscles in WT (2.1 ± 2.5%, *P *<* *0.05 by Tukey's test, *n *=* *6) (Fig. 2G). Importantly, in Epac1KO, the response to CB of all muscles examined in this study was suppressed to a similar extent (TA: 1.3 ± 5.4%; SOL: 1.3 ± 5.5%; MA: −5.6 ± 1.6%; cardiac muscle: 0.1 ± 4.0%, *P *= NS by Tukey's test, *n *=* *5–6) (Fig. 2H).

These data suggest that activation of Epac1‐regulated hypertrophic signaling following *β*_2_‐AR stimulation is essential for CB‐induced muscle hypertrophy, and thus the decreased efficiency of CB for inducing SOL muscle hypertrophy in WT might be consequence of impaired Epac1‐mediated hypertrophic signaling.

### MHC isoform composition was not altered in TA muscle of Epac1KO

The average MHC isoform composition in TA obtained from Control and CB‐treated WT or Epac1KO by SDS‐PAGE analysis (Fig. 3A). TA primarily contains MHC‐IId/x and MHC‐IIb, and their average compositions were not significantly different between WT and Epac1KO at baseline (IId/x: 29 ± 2.6% (WT) vs. 34 ± 3.0% (Epac1KO); IIb: 71 ± 2.6% (WT) vs. 66 ± 3.0% (Epac1KO), *P *<* *0.05 by Tukey's test, *n *=* *6 each) (Fig. 3A, significant genotype and treatment main effects in MHC‐IId/x and MHC‐IIb, *P *<* *0.01 and *P *<* *0.05, respectively, by two‐way ANOVA). Similarly, there was no significant difference after CB treatment (IId/x: 22 ± 2.2% (WT) vs. 31 ± 1.3% (Epac1KO); IIb: 78 ± 2.2% (WT) vs. 70 ± 1.3% (Epac1KO), *P *= NS by Tukey's test, *n *=* *6 each).

These data suggest that Epac1 signaling has no effect on the MHC isoform composition of TA muscle in either the Control or CB‐treated groups.

### MHC isoform transition toward slower isoforms was induced in SOL muscle of Epac1KO

The average MHC isoform composition in SOL from Control and CB‐treated WT and Epac1KO mice was examined by SDS‐PAGE analysis (Fig. 3B, significant genotype main effect, *P *<* *0.001 in MHC‐1 and MHC‐IIb and *P *<* *0.01 in MHC‐IId/x by two‐way ANOVA). SOL primarily contains MHC‐I and MHC‐IIa, in addition to MHC‐IId/x and a small population of MHC‐IIb (Fig. 3B *upper*). The average proportion of MHC‐I was significantly greater in Epac1KO compared with WT in both the Control and CB‐treated groups (Control: 31 ± 1.8% (WT) vs. 40 ± 2.2% (Epac1KO), *P *<* *0.05 by Tukey's test, *n *=* *6 each; CB: 28 ± 1.9% (WT) vs. 38 ± 1.9% (Epac1KO), *P *<* *0.01 by Tukey's test, *n *=* *6 each). On the other hand, the average proportion of IIb was significantly decreased in Epac1KO, compared with WT in both the Control and CB‐treated groups (Control: 15 ± 0.9% (WT) vs. 9.8 ± 0.9% (Epac1KO), *P *<* *0.01 by Tukey's test, *n *=* *6 each; CB: 16 ± 1.1% (WT) vs. 10 ± 0.9% (Epac1KO), *P *<* *0.01 by Tukey's test, *n *=* *6 each). The average proportions of MHC‐IIa and MHC‐IId/x were not significantly different between WT and Epac1KO in either the Control or CB‐treated groups (*P *= NS by Tukey's test, *n *=* *6). These data suggest that MHC isoform transition toward slower isoforms was induced in SOL of Epac1KO.

### Histochemical staining of type I and type II fibers in SOL muscle

In order to confirm the results of SDS‐PAGE analysis, we performed histochemical staining for type I and type II myosin in SOL of WT and Epac1KO (Fig. 3C). The proportion of type I fibers in Epac1KO was significantly greater than that in WT in both the Control and CB‐treated groups (Control: 30 ± 1.8% (WT) vs. 39 ± 1.7% (Epac1KO)*, P *<* *0.01 by Tukey's test, *n *=* *6; CB: 29 ± 1.7% (WT) vs. 39 ± 1.4% (Epac1KO), *P *<* *0.01 by Tukey's test, *n *=* *6), while the proportion of type II fibers in Epac1KO was significantly smaller than that in WT in both the Control and CB‐treated groups (Control: 65 ± 2.1% (WT) vs. 57 ± 1.3% (Epac1KO)*, P *<* *0.05 by Tukey's test, *n *=* *6; CB: 68 ± 2.0% (WT) vs. 56 ± 1.6% (Epac1KO), *P *<* *0.01 by Tukey's test, *n *=* *6) (Fig. 3D, significant genotype main effect in type 1 and type II, *P *<* *0.001 by two‐way ANOVA). The proportion of intermediate type (Type I/II), which reacts with type I and type II myosin antibodies, was not significantly different between the Control and CB‐treated WT or Epac1KO (Control: 4.5 ± 1.3% (WT) vs. 3.8 ± 0.7% (Epac1KO)*, P *= NS by Tukey's test, *n *=* *6; CB: 3.0 ± 0.7% (WT) vs. 5.4 ± 0.6% (Epac1KO), *P *= NS by Tukey's test, *n *=* *6) (Fig. 3D, genotype and treatment main effects, and interaction effect in type I/II, *P *= NS by two‐way ANOVA).

These results confirm that MHC isoform transition toward slower isoforms occurred in SOL muscle of Epac1KO, in agreement with the SDS‐PAGE analysis (Fig. 3B).

### CB‐mediated Akt/mTOR pathway activation in TA muscle was attenuated in Epac1KO

We have recently demonstrated that chronic *β*_2_‐AR stimulation with CB activates the Akt/mTOR pathway, a major hypertrophic signaling pathway for skeletal muscle, in masseter muscle of WT, but this activation was suppressed in Epac1KO (Ohnuki et al. 2014). Here, we observed CB‐mediated skeletal muscle hypertrophy in TA (fast‐twitch) muscle of WT, in agreement with the previous finding in masseter muscle, but not in SOL (slow‐twitch) muscle (Fig. 2).

In order to examine the mechanism of the muscle‐specific hypertrophic response to CB, we first examined Akt phosphorylation at serine 473 (Fig. 4A, significant treatment main effect and interaction effect, *P *<* *0.05 by two‐way ANOVA) in TA prepared from WT and Epac1KO and found that it was significantly increased by CB in WT, but not in Epac1KO (WT: from 100 ± 16% to 242 ± 45%, *P *<* *0.05 vs. Control by Tukey's test, *n *=* *5; Epac1KO: from 165 ± 12% to 155 ± 2%, *P *= NS vs. Control by Tukey's test, *n *=* *5).

We also examined activation of the Akt/mTOR pathway in terms of S6K1 phosphorylation on threonine 389 after CB treatment (Fig. 4B, significant treatment main effect and interaction effect, *P *<* *0.05 and *P *<* *0.01, respectively, by two‐way ANOVA) and found that this phosphorylation was significantly increased in WT (from 100 ± 7.4% to 139 ± 9.0%, *P *<* *0.01 vs. Control by Tukey's test, *n *=* *6), but not in Epac1KO (from 110 ± 7.9% to 106 ± 2.2%, *P *= NS vs. Control by Tukey's test, *n *=* *6).

These data suggest that Epac1 is required for the development of hypertrophy of TA, as in the case of masseter muscle (Ohnuki et al. 2014), suggesting that activation of the Akt/mTOR pathway may play a general role in CB‐induced fast‐twitch muscle hypertrophy.

### CB‐mediated CaMKII/HDAC4 pathway activation in TA muscle was attenuated in Epac1KO

Phosphorylation of HDAC4 on serine 256/266 mediated by CaMKII was significantly increased in masseter muscle of WT, but not in Epac1KO (Ohnuki et al. 2014). We thus examined the phosphorylation of CaMKII on threonine 286 (Fig. 4C, significant treatment main effect and interaction effect, *P *<* *0.05 by two‐way ANOVA) and HDAC4 on serine 246 (Fig. 4D, significant treatment main effect and interaction effect, *P *<* *0.001 and *P *<* *0.05, respectively, by two‐way ANOVA) in TA prepared from WT or Epac1KO, and found that they were significantly increased in WT (CaMKII: from 100 ± 20.8% to 195 ± 27.5%, *P *<* *0.05 vs. Control by Tukey’ s test, *n *=* *5–6; HDAC4: from 100 ± 8.6% to 163 ± 11.5%, *P *<* *0.01 vs. Control by Tukey's test, *n *=* *6), but not in Epac1KO (CaMKII: from 121 ± 12.7% to 130 ± 10.1%, *P *= NS vs. Control by Tukey's test, *n *=* *4–6; HDAC4: from 120 ± 5.6% to 134 ± 8.2%, *P *= NS vs. Control by Tukey's test, *n *=* *6). These data suggest that the CaMKII/HDAC4 pathway, as well as the Akt/mTOR pathway, might be important for the development of hypertrophy in both TA and masseter muscle.

### CB did not activate the Akt/mTOR pathway in SOL muscle of either WT or Epac1KO

We next examined the phosphorylation of Akt/mTOR pathway in SOL in Control and CB‐treated WT and Epac1KO (Fig. 5A and B).

Chronic CB treatment did not significantly increase Akt phosphorylation on serine 473 from baseline in SOL of either WT or Epac1KO (WT: from 100 ± 13.4% to 121 ± 12.1%; Epac1KO: from 134 ± 13.4% to 116 ± 23%, *P *= NS vs. Control by Tukey's test, *n *=* *6) (Fig. 5A, genotype and treatment main effects, and interaction effect, *P *= NS by two‐way ANOVA). Also, it did not significantly increase the phosphorylation of S6K1 on threonine 389 from baseline in either WT or Epac1KO (WT: from 100 ± 15.8% to 93 ± 19.1%; Epac1KO: from 92 ± 16.5% to 108 ± 19.0%, *P *= NS vs. Control by Tukey's test, *n *=* *6 each) (Fig. 5B, genotype and treatment main effects, and interaction effect, *P *= NS by two‐way ANOVA). These data suggest that the failure of CB to induce SOL muscle hypertrophy in WT is due to loss of Akt/mTOR pathway activation, independently of Epac1 expression.

### CB did not activate the CaMKII/HDAC4 pathway in SOL muscle in either WT or Epac1KO

We also examined the activation of CaMKII/HDAC4 pathway in SOL of Control and CB‐treated WT and Epac1KO (Fig. 5C and D).

CaMKII phosphorylation on threonine 286 was not significantly increased from baseline in either WT or Epac1KO after CB treatment (WT 100 ± 13.8% (Control) vs. 125 ± 17.0% (CB); Epac1KO 136 ± 12.6% (Control) vs. 102 ± 11.7% (CB), *P *= NS by Tukey's test, *n *=* *6 each) (Fig. 5C, significant interaction effect, *P *<* *0.05 by two‐way ANOVA). Also, HDAC4 phosphorylation on serine 246 was not significantly increased from baseline in either WT or Epac1KO after CB treatment (WT: from 100 ± 2.3% to 85 ± 12.6%; Epac1KO: from 88 ± 8.8% to 117 ± 10.8%, *P *= NS vs. Control by Tukey's test, *n *=* *6 each) (Fig. 5D, significant interaction effect, *P *<* *0.05 by two‐way ANOVA).

These data, together with the data in Figure 5A and B, suggest that CB does not activate Epac1 or its downstream hypertrophic signaling, that is, the Akt/mTOR pathway and CaMKII/HDAC4 pathways in SOL in either WT or Epac1KO, supporting the idea that loss of activation of downstream hypertrophic signaling might be the reason for the lack of hypertrophic activity of CB in SOL of WT.

### Expression of *β*_2_‐AR, Epac1/2, PDE4, and PKA in TA and SOL

In order to examine the reason for the lack of efficiency of CB in SOL, we measured the expression of *β*_2_‐AR, Epac1, Epac2, PDE, and PKA in TA and SOL prepared from WT and Epac1KO by means of immunoblotting (Fig. 6A) (Okumura et al. 2003a,b, 2007; Kamide et al. 2015). Although 11 distinct families of PDEs have been defined so far, PDE4 accounts for >80% of total PDE activity in skeletal muscle (Bloom 2002; Hinkle et al. 2005). Also, PDE4 proteins are encoded by four genes (PDE4A, PDE4B, PDE4C, and PDE4D) in mammals, and PDE4D is important to maintain the resting cAMP level through its negative regulatory effect on cAMP/Epac/Akt signaling in skeletal muscle (McCahill et al. 2008; Joshi et al. 2014). We thus examined the PDE4D expression in terms of PDE4D long isoforms (PDE4D‐L) (including PDE4D3 (76 kDa), PDE4D4 (91 kDa), and PDE4D5 (84 kDa)) and short isoforms (PDE4D‐S (including PDE4D1 (66 kDa) and PDE4D2 (58 kDa)) (Bolger et al. 1997; Cheung et al. 2007). In WT, expressions levels of *β*_2_‐AR, Epac1, Epac2, PDE4D‐L, PDE4D‐S, and PKA in SOL were significantly greater than those in TA (*β*_2_‐AR: 7.6 ± 1.7, Epac1: 5.1 ± 0.7, Epac2: 2.8 ± 0.4, PDE4D‐L: 12 ± 1.5, PDE4D‐S: 1.7 ± 0.2, PKA: 3.3 ± 0.5 fold, *P *<* *0.01 in *β*_2_‐AR, PDE4D‐S and PKA and *P *<* *0.001 in Epac1, Epac2 and PDE4D‐L vs. TA by unpaired *t*‐test, *n *=* *4–5 each) (Fig. 6B). In Epac1KO, expression levels in SOL were similarly and significantly greater than those in TA muscle prepared from Epac1KO (*β*_2_‐AR: 11.2 ± 1.1, Epac2: 2.8 ± 0.2, PDE4D‐L: 10.1 ± 1.0, PDE4D‐S: 1.8 ± 0.1, PKA: 4.2 ± 0.2 fold, *P *<* *0.001 vs. TA by unpaired *t*‐test, *n *=* *4–5 each) (Fig. 6C). Importantly, the fold increase of PDE4D‐L, which includes PDE4D5, was much greater than that of other cAMP signaling components. These data, taken together with the previous report of preferential coupling of PDE4D5 with *β*_2_‐AR (Berthouze‐Duquesnes et al. 2013), suggest that cAMP hydrolysis mediated via PDE4D is increased in SOL and this is likely to result in a lower tissue concentration of cAMP.

### CB‐mediated increase of cAMP levels was attenuated in SOL muscle of both WT and Epac1KO

The cAMP levels in TA and SOL of WT and Epac1KO were examined at 1 hour after a single injection of CB (2 mg/kg, i.p.) or vehicle alone.

In WT, cAMP level was significantly increased in TA (Control vs. CB: 441 ± 39 fmol/mg vs. 635 ± 35 fmol/mg, *P *<* *0.01 by unpaired *t*‐test, *n *=* *6–7), but the increase was suppressed in SOL muscle (Control vs. CB: 480 ± 35 fmol/mg vs. 525 ± 41 fmol/mg, *P *= NS by unpaired *t*‐test, *n *=* *6–7) (Fig. 7A). In Epac1KO, cAMP level was also significantly increased in TA (Control vs. CB: 457 ± 28 fmol/mg vs. 670 ± 20 fmol/mg, *P *<* *0.001 by unpaired *t*‐test, *n *=* *6), and again the increase was suppressed in SOL (Control vs. CB: 594 ± 29 fmol/mg vs. 640 + 26 fmol/mg, *P *= NS by unpaired *t*‐test, *n *=* *6) (Fig. 7A).

These data indicated that cAMP production in response to CB was attenuated in SOL muscle, compared to that in TA muscle in both WT and Epac1KO.

### CB‐induced PKA and CREB phosphorylation in TA and SOL

In order to confirm the differential effects of CB on cAMP production in TA and SOL, we examined activation of the cAMP/PKA pathway by measuring phosphorylation of PKA‐catalytic unit on threonine 198 and cAMP response element binding protein (CREB) on serine 133 in TA and SOL of WT and Epac1KO after chronic CB infusion for 3 weeks (Fig. 7B–D).

In TA muscle, phosphorylation of PKA‐catalytic unit (threonine 198) was significantly increased in the CB‐treated group in both WT (100 ± 9.7% (Control) vs. 145 ± 9.8% (CB), *P *<* *0.05 by unpaired *t*‐test, *n *=* *5) and Epac1KO (100 ± 8.4% (Control) vs. 153 ± 15% (CB), *P *<* *0.05 by unpaired *t*‐test, *n *=* *5). However, in SOL muscle, the increase was suppressed in both WT (100 ± 11% (Control) vs. 84 ± 4.5% (CB), *P *= NS by unpaired *t*‐test, *n *=* *5–6) and Epac1KO (100 ± 9.8% (Control) vs. 117 ± 9.5% (CB), *P* = NS by unpaired *t*‐test, *n *=* *6) (Fig. 7B and D).

As in the case of PKA‐catalytic unit, phosphorylation of CREB (serine 133) was significantly increased in both WT (100 ± 11.6% (Control) vs. 164 ± 16.8% (CB), *P *<* *0.01 by unpaired *t*‐test, *n *=* *5–6) and Epac1KO (100 ± 6.7% (Control) vs. 155 ± 8.9% (CB), *P *<* *0.01 by unpaired *t*‐test, *n *=* *4–5). However, in SOL muscle, the increase was again suppressed in both WT (100 ± 14.5% (Control) vs. 94 ± 9.9% (CB), *P *= NS by unpaired *t*‐test, *n *=* *5–6) and Epac1KO (100 ± 17.6% (Control) vs. 89 ± 11.4% (CB), *P *= NS by unpaired *t*‐test, *n *=* *6) (Fig. 7C and D).

These data, together with the data shown in Figure 7A, suggested that production of cAMP in response to CB was attenuated in SOL, compared to that in TA, and this might be associated with decreased activation of hypertrophic signaling, such as the cAMP/Epac/Akt/mTOR and cAMP/Epac1/CaMKII/HDAC4 pathways in SOL.

## Discussion

A severe loss of muscle mass is a risk factor for mortality in a number of conditions and disease states. Loss of protein from skeletal muscle fibers can lead to severe and progressive muscle wasting, that is, muscle atrophy and weakness, including death due to Duchenne muscular dystrophy (Wicklund 2013), and it is also involved in other conditions, including chronic obstructive pulmonary disease, cancer‐associated cachexia, diabetes, renal failure, cardiac failure, Cushing syndrome, sepsis, burns, and trauma (Cohen et al. 2015; Shiozawa et al. 2016).

Synthetic *β*_2_‐AR agonists such as CB were developed primarily to facilitate dilatation of the bronchiolar smooth muscle in asthma patients (Ball et al. 1991). However, it became apparent that *β*_2_‐AR agonists caused an increase in body mass at higher doses, which was later attributed to their powerful anabolic activity and consequent increase in skeletal muscle mass (Emery et al. 1984). Not surprisingly, numerous studies have focused on therapeutic applications of CB for ameliorating muscle wasting and for improving muscle function in disorders such as muscular dystrophy (Kissel et al. 1998; Fowler et al. 2004; Umeki et al. 2015) and heart failure (Birks et al. 2011). On the other hand, studies on animals have shown that CB impairs heart and skeletal muscle function, including tachycardia, cardiac hypertrophy, and decreased cardiac performance (Hoey et al. 1995; Ohnuki et al. 2013). It is also reported that CB shows muscle selectivity and its anabolic effect seems greater in fast‐twitch muscle than in slow‐twitch skeletal muscle, though the mechanisms involved are unclear (Reeds et al. 1986; Ryall et al. 2002; Sirvent et al. 2014).

Unlike other adrenergic agents such as isoproterenol (a nonselective *β*‐AR agonist) (Yin et al. 2015), pharmacological stimulation of *β*_2_‐AR with CB induces hypertrophy of TA (fast‐twitch), masseter muscle (fast‐twitch), and cardiac muscle, but not SOL (slow‐twitch) in WT, without causing an increase in interstitial collagen (fibrosis) as shown in this study (Fig. 2) and previously by other groups (Wong et al. 1997, 1998; Zeman et al. 2000), even though mice with a very high level of overexpression of *β*_2_‐AR develop fibrosis (Liggett et al. 2000). Since CB is known to promote lipolysis and decrease fat tissue, we speculate that those changes might compensate at least in part for the increase of the skeletal muscle mass, resulting in no significant difference of total BW between the Control and CB‐treated groups (Miller et al. 1988; McElligott et al. 1989; Moore et al. 1994; Abo et al. 2012; Ohnuki et al. 2013).

**Figure 8:**
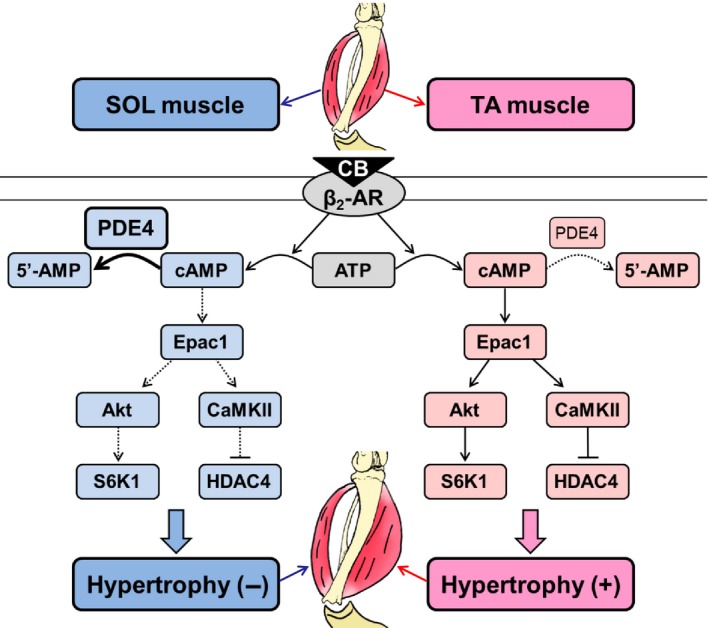
Schematic summary of the proposed mechanism of the suppressed hypertrophic response to CB in SOL (*left*), compared to TA muscle (*right*). This scheme illustrates the proposed relationship between PDE4 expression and Epac1‐mediated skeletal muscle hypertrophy via activation of Akt/mTOR and CaMKII/HDAC4 signaling. PDE4 expression in SOL muscle (*left*) is greater than that in TA (*right*) and this is considered to result in increased cAMP hydrolysis, and thus a lower tissue concentration of cAMP, which may be insufficient to induce activation of hypertrophic signaling mediated by Epac1 and its downstream Akt/S6K1 and CaMKII/HDAC4 signaling pathways in SOL muscle.

Therefore, the primary objective of this study was to investigate the molecular mechanisms of the differential anabolic effects of CB on SOL (slow‐twitch) muscle and TA (fast‐twitch) muscle. Differences in muscle responsiveness to CB do not simply reflect differences in *β*_2_‐AR density, as this is greater in slow‐twitch muscle than in fast‐twitch muscle, as demonstrated in this study (Fig. 8) and in previous work (Ryall et al. 2002). We thus anticipated that CB‐induced activation of hypertrophic signaling downstream of *β*_2_‐AR might be attenuated in slow‐twitch muscle, compared to that in fast‐twitch muscle.

Recently, we demonstrated that Epac1‐mediated activation of the Akt/mTOR and CaMKII/HDAC4 pathways was attenuated in CB‐treated masseter muscle of Epac1KO, and this appeared to account for the loss of masseter muscle hypertrophy in Epac1KO in response to CB treatment (Ohnuki et al. 2014). Therefore, we next examined activation of the Akt/mTOR pathway and CaMKII/HDAC4 pathways in TA and SOL prepared from Control and CB‐treated WT and Epac1KO. These pathways were significantly activated from baseline by CB (2 mg/kg/day for 3 weeks) in TA from WT, but not in Epac1KO, as previously reported for masseter muscle (Ohnuki et al. 2014). On the other hand, CB‐mediated activation of these signaling pathways was attenuated in SOL from both Epac1KO and WT. We thus anticipated that Epac1 expression and/or Epac1 activation by cAMP might not be sufficient for activation of downstream signaling in SOL muscle after CB treatment. However, it should be noted that relatively small number of animal were used in this work, and we cannot rule out the possibility that the statistical power of this study was insufficient to detect a CB‐mediated hypertrophic effect on SOL muscle or activation of downstream signaling in SOL after CB treatment.

We thus measured Epac1 expression in TA and SOL muscles by immunoblotting and unexpectedly found that Epac1 expression was increased by approximately fivefold in SOL, compared to TA. We next considered that cAMP might be decreased in SOL, compared to TA. In order to test this hypothesis, we examined PDE4 expression because PDE4 contributes predominantly to cAMP hydrolysis in skeletal muscle, accounting for more than 80% of the PDE activity in human and rodent skeletal muscle (Bloom 2002; Hinkle et al. 2005). Also, PDE4D expression was reported to serve as a major modulator of intracellular cAMP levels in skeletal muscle (Lania et al. 1998; McCahill et al. 2008; Joshi et al. 2014). More importantly, PDE inhibition with torbafylline, a nonselective PDE inhibitor, attenuates burn‐induced skeletal muscle atrophy through the PDE4/cAMP/Epac/phosphoinositol 3‐kinase (PI3K)/Akt/mTOR pathway in vivo (Joshi et al. 2014).

We found that protein expression of PDE4 (long isoforms) in SOL was significantly greater than that in TA in both WT and Epac1KO. Based on this and previous findings, we considered that the increased PDE4 expression in SOL might reduce the cAMP concentration to a level that is insufficient to activate the Akt/mTOR and CaMKII/HDAC4 pathways, thereby accounting for the failure of CB to induce hypertrophy of this slow‐twitch muscle.

Other possible mechanisms contributing to the negative hypertrophic effect of CB on SOL muscle are induction of myocyte apoptosis and inhibition of the ubiquitin–proteosome pathway, which were reported to be more pronounced in slow‐twitch muscle (SOL) than in fast‐twitch muscle (TA) (Burniston et al. 2005; Yimlamai et al. 2005; Douillard et al. 2011).

The *β*‐AR signaling pathway is considered a therapeutic target for the treatment of skeletal muscle wasting and weakness due to its critical role in the mechanisms controlling protein synthesis and degradation, in addition to the modulation of muscle fiber type (Ohnuki et al. 2013, 2014; Umeki et al. 2013, 2015). Older generation *β*_2_‐agonists, such as CB or fenoterol, are powerful muscle anabolic agents when administered to rats at relatively high (mg/kg) doses, but elicit a markedly lesser effect when administered at what would be considered therapeutic doses (*μ*g/kg), such as the doses employed in human (asthmatic) patients and other species (e.g., horses) for the management of inflammatory airway disease (Plant et al. 2003; Malinowski et al. 2004). Otherwise, CB administered to rats at a low dose of 10 *μ*g/kg/day had only modest effects on slow‐twitch skeletal muscle and no discernable effect on fast‐twitch skeletal muscles (Chen and Alway 2000).

We believe our current experimental data will be helpful in developing pharmacological approaches to the treatment of skeletal muscle wasting and weakness with new generation *β*‐agonists, which would be able to elicit an anabolic response in skeletal muscle while exhibiting reduced effects on muscle selectivity and the cardiovascular systems, compared with older generation *β*‐agonists such as CB and fenoterol (Lynch and Ryall 2008).

## Conflict of Interest

None declared.
